# Supplementary material for: Pretreatment Neutrophil-to-Lymphocyte Ratio as a Prognostic Marker for the Outcome of HPV-Positive and HPV-Negative Oropharyngeal Squamous Cell Carcinoma
Source: Viruses. 2023 Jan 10;15(1):198. doi: 10.3390/v15010198 (PMC9863220; doi:10.3390/v15010198)

Figure S1. Flowchart of patient exclusion from the database population to the final included study population.

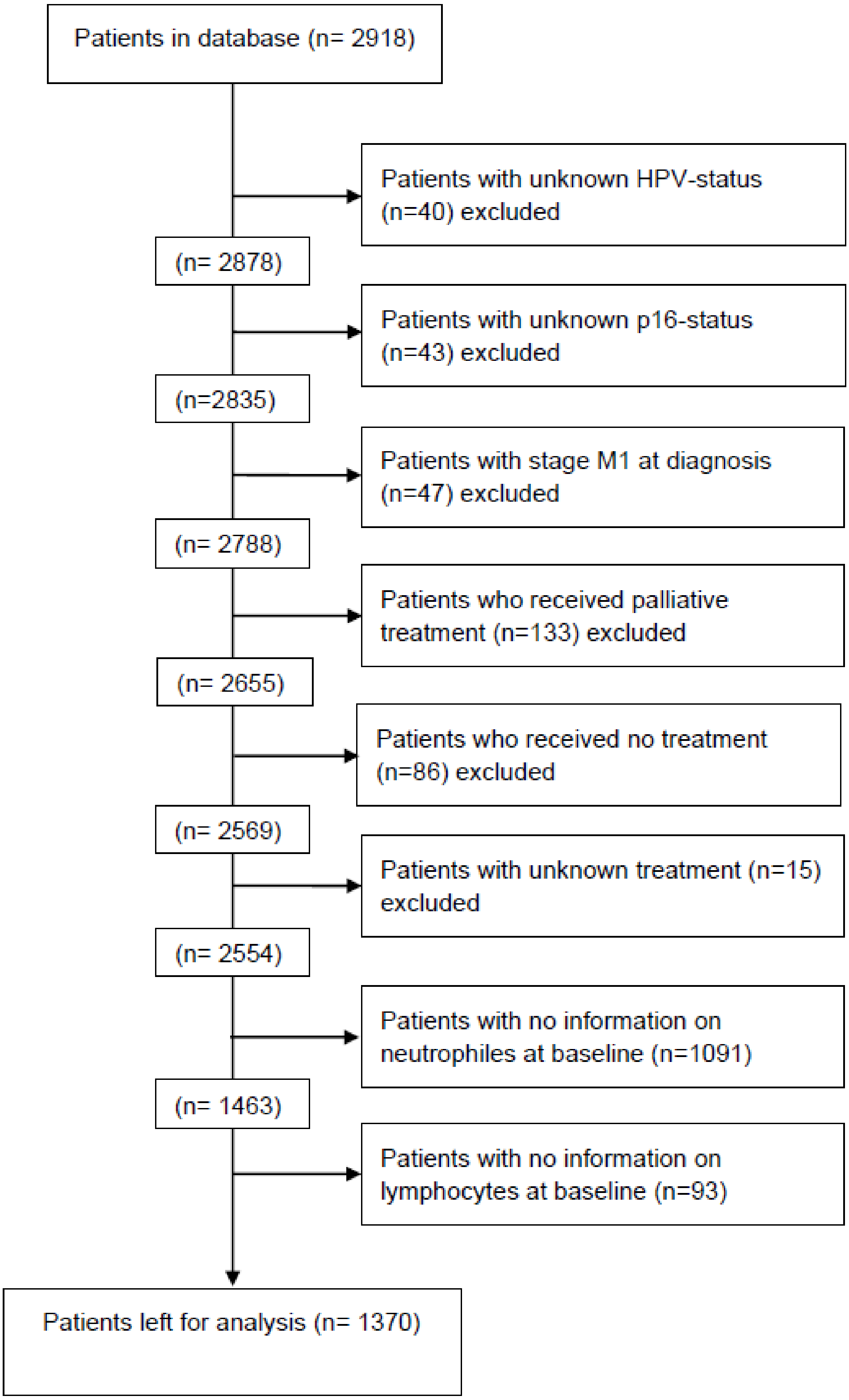

Supplement: Supplementary file 1 [file viruses-15-00198-s001.zip › Figure S1, Viruses, Supplementary Materials.pdf]
