# Supplementary figures and images for: Pretreatment Neutrophil-to-Lymphocyte Ratio as a Prognostic Marker for the Outcome of HPV-Positive and HPV-Negative Oropharyngeal Squamous Cell Carcinoma
Source: Viruses. 2023 Jan 10;15(1):198. doi: 10.3390/v15010198 (PMC9863220; doi:10.3390/v15010198)

Figure S2. Proportional Hazards test with Schoenfeld residuals for categorical covariates.

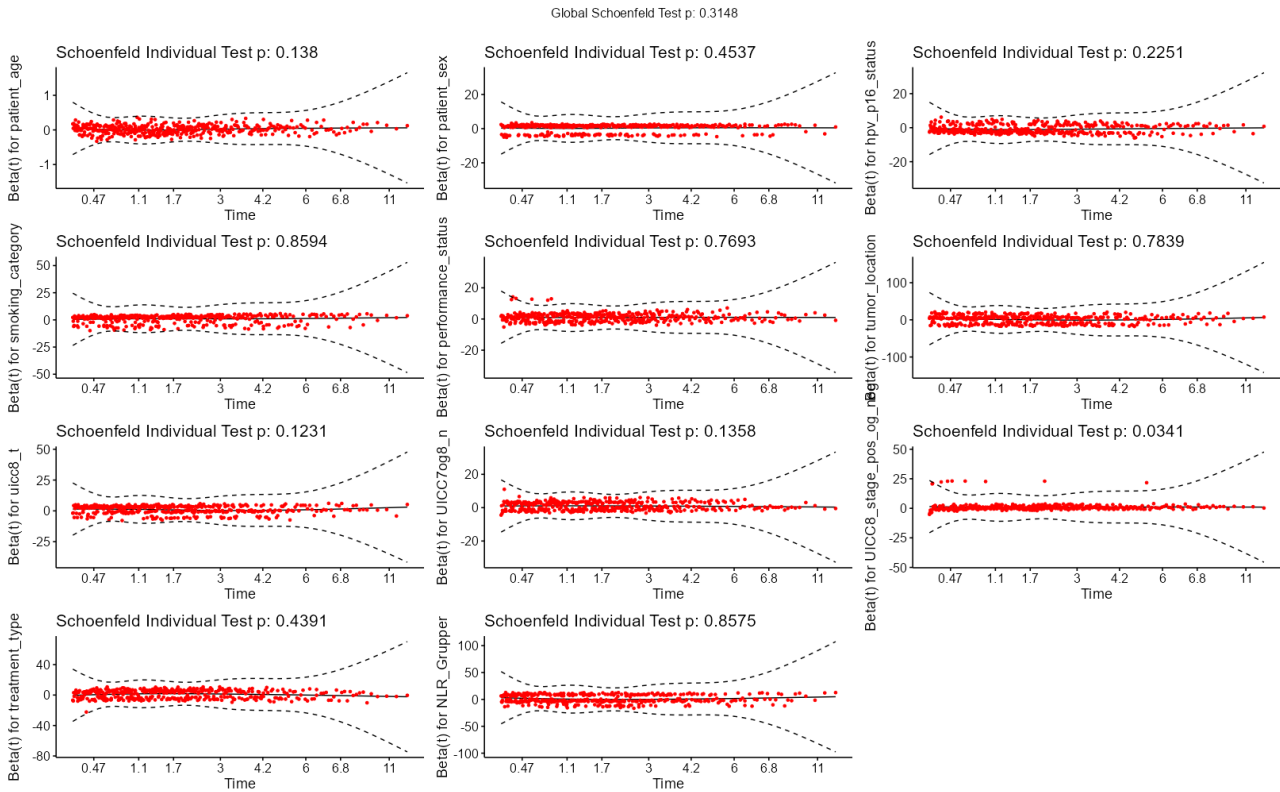

Supplement: Supplementary file 1 [file viruses-15-00198-s001.zip › Figure S2, Viruses, Supplementary Materials.pdf]

**Figure S3.** Non-linearity test with Martingale Residual of continuous covariate patient age.

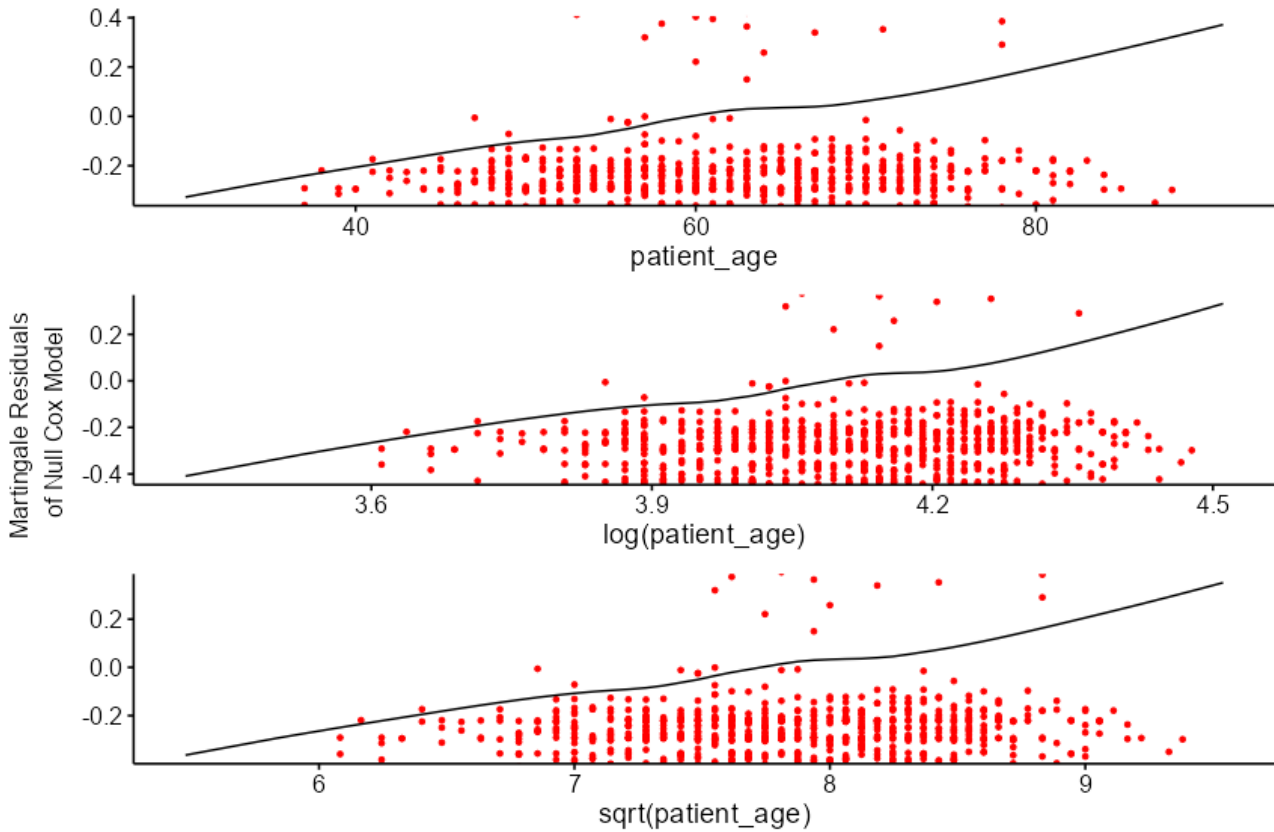

Supplement: Supplementary file 1 [file viruses-15-00198-s001.zip › Figure S3, Viruses, Supplementary Materials.pdf]
